# Cardiac regeneration by direct reprogramming in this decade and beyond

**DOI:** 10.1186/s41232-021-00168-5

**Published:** 2021-07-01

**Authors:** Hiroyuki Yamakawa, Masaki Ieda

**Affiliations:** 1 Department of Cardiology, Keio University School of Medicine, 35 Shinanomachi, Shinjiku-ku, Tokyo, 160-8582 Japan; 2 Center for Preventive Medicine, Keio University School of Medicine, Tokyo, Japan; 3 Department of Cardiology, Faculty of Medicine, University of Tsukuba, 1-1-1 Tennoudai, Tsukuba City, Ibaraki, 305-8575 Japan

**Keywords:** Induced cardiomyocytes (iCMs), Direct cardiac reprogramming, iPS cells, Cardiac fibroblast, Transcription factors, Micro RNAs, Sendai virus

## Abstract

Japan faces an increasing incidence of heart disease, owing to a shift towards a westernized lifestyle and an aging demographic. In cases where conventional interventions are not appropriate, regenerative medicine offers a promising therapeutic option. However, the use of stem cells has limitations, and therefore, “direct cardiac reprogramming” is emerging as an alternative treatment. Myocardial regeneration transdifferentiates cardiac fibroblasts into cardiomyocytes in situ.

Three cardiogenic transcription factors: Gata4, Mef2c, and Tbx5 (GMT) can induce direct reprogramming of fibroblasts into induced cardiomyocytes (iCMs), in mice. However, in humans, additional factors, such as Mesp1 and Myocd, are required. Inflammation and immune responses hinder the reprogramming process in mice, and epigenetic modifiers such as TET1 are involved in direct cardiac reprogramming in humans. The three main approaches to improving reprogramming efficiency are (1) improving direct cardiac reprogramming factors, (2) improving cell culture conditions, and (3) regulating epigenetic factors. miR-133 is a potential candidate for the first approach. For the second approach, inhibitors of TGF-β and Wnt signals, Akt1 overexpression, Notch signaling pathway inhibitors, such as DAPT ((S)-tert-butyl 2-((S)-2-(2-(3,5-difluorophenyl) acetamido) propanamido)-2-phenylacetate), fibroblast growth factor (FGF)-2, FGF-10, and vascular endothelial growth factor (VEGF: FFV) can influence reprogramming. Reducing the expression of Bmi1, which regulates the mono-ubiquitination of histone H2A, alters histone modification, and subsequently the reprogramming efficiency, in the third approach. In addition, diclofenac, a non-steroidal anti-inflammatory drug, and high level of Mef2c overexpression could improve direct cardiac reprogramming.

Direct cardiac reprogramming needs improvement if it is to be used in humans, and the molecular mechanisms involved remain largely elusive. Further advances in cardiac reprogramming research are needed to bring us closer to cardiac regenerative therapy.

## Introduction

In Japan, the incidence of ischemic heart diseases is increasing, due to the adoption of a westernized lifestyle, and an increase in hypertension and valvular diseases due to an aging population. This situation has made cardiac disease the second most common cause of death, after cancer. The heart is composed of different types of cells, and cardiac function is carefully regulated, not only by cardiomyocytes, but also by other cells, such as vascular endothelial cells and fibroblasts. Cardiomyocytes account for approximately 30% of all cells in the heart, and at least 50% of the remaining cells are non-cardiomyocytes [1, 2]. Cardiomyocytes are terminally differentiated cells with no potential for self-renewal; cardiomyocytes that become necrotic due to myocardial infarction, heart failure, or other cardiac diseases are therefore replaced by proliferating fibroblasts. This situation results in scarring of the affected site due to the formation of fibrotic tissue. These fibrotic changes reduce the cardiac systolic function, and arrhythmia caused by scar tissue has a poor prognosis. Heart transplantation is the last resort for serious cardiac failure with no expectation of improvement through optimal pharmacological or non-pharmacological therapies. However, a shortage of donors in Japan, transplant rejection, costs, and other factors limit widespread transplantation, and therefore, the field of cardiac regenerative medicine is attracting significant interests. One promising approach to cardiac regeneration is to differentiate stem cells, such as induced pluripotent stem cells (iPS cells) into cardiomyocytes outside the body, and then transplant the differentiated cardiomyocytes into the body. However, generating the large numbers of cells required to replace as many as 1 billion cardiomyocytes lost to myocardial infarction or failing heart incurs enormous costs. It also poses other limitations, such as the presence of residual stem cells undergoing oncogenesis, and a low survival rate of transplanted cells [3].

**Fig. 1:**
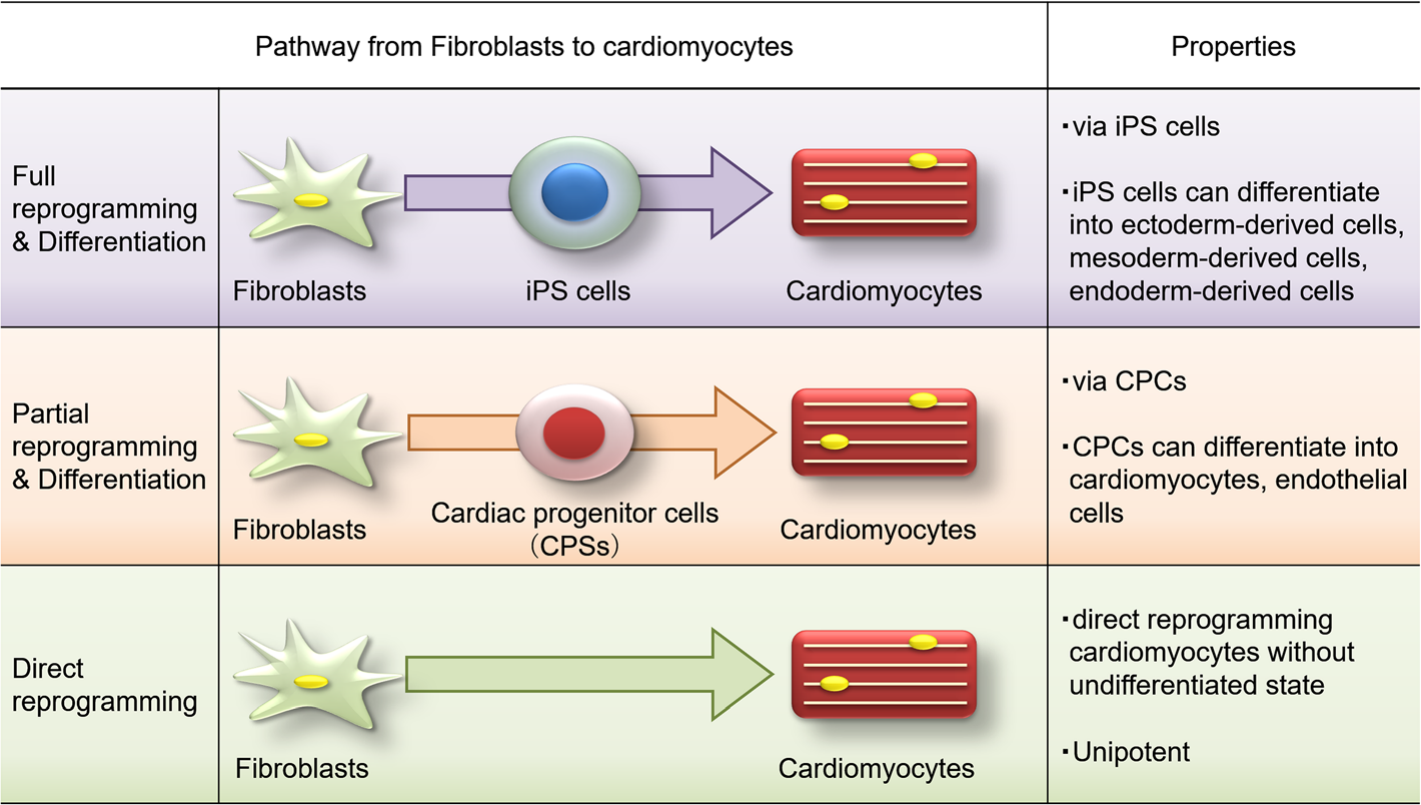
Three pathways for deriving cardiomyocytes for myocardial regeneration [4]. There are (1) full reprogramming approaches (purple line), (2) partial reprogramming approaches (orange line), and (3) direct reprogramming approaches (green line). For the treatment of myocardial infarction and heart failure, (1) iPS-derived cardiomyocytes are about to be clinically studied for myocardial transplantation experiments. (3) Direct cardiac reprogramming does not require cardiomyocyte transplantation and may be feasible through a direct reprogramming approach. (2) Partial reprogramming may offer the advantages of (1) and (3). Further research is desirable in the future

The discovery of iPS cells suggests that terminally differentiated cells can be changed into other cells by specific transcription factors. In 2010, we reported a novel strategy for the direct reprogramming of fibroblasts into cardiomyocytes. Based on these results, there are currently three possible pathways for the creation of cardiac muscle from fibroblasts. The three pathways can be summarized as follows (Fig.1): (1) full reprogramming of fibroblasts into iPS cells and subsequent cardiac differentiation, (2) partial reprogramming of fibroblasts into cardiac progenitor cells and subsequent differentiation, and (3) direct reprogramming of fibroblasts into cardiomyocytes [4–6].

**Fig. 2:**
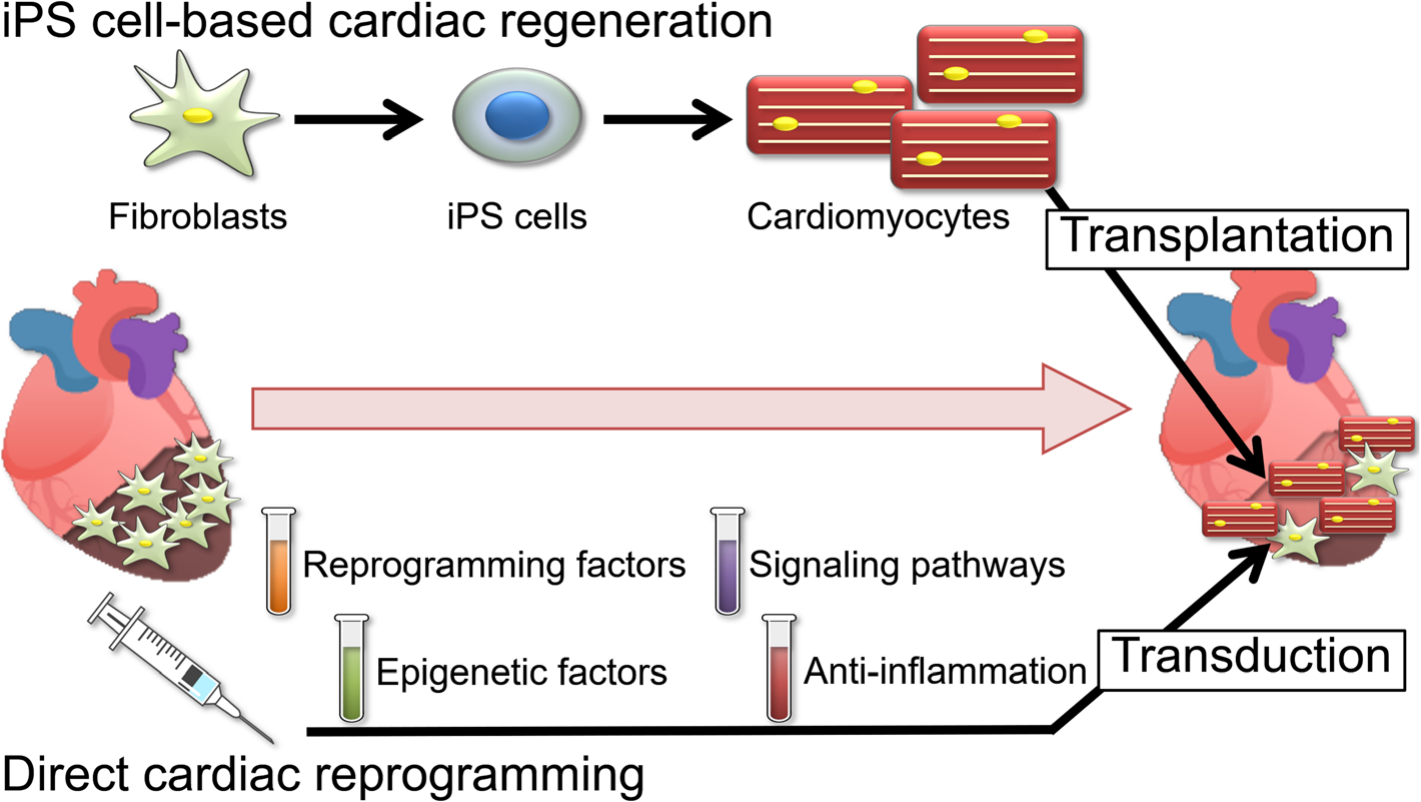
Schema of future heart regenerative medicine. The outline of heart regenerative medicine in the case of myocardial infarction is shown. When the heart is damaged due to myocardial infarction, heart failure, and so on, cardiomyocytes are preferentially lost. The fibroblasts of the heart remain and replace the lost cardiomyocytes. Currently, a method of transplanting iPS-derived cardiomyocytes is becoming a reality (upper side). Another method is the transduction of the cardiomyocytes based on the remaining fibroblasts. This is the direct cardiac reprogramming (lower side)

As mentioned earlier, we proposed the concept of “direct cardiac reprogramming” in place of this conventional method of cell transplantation. This is a technique that converts cardiac fibroblasts, which are present in large numbers in the myocardium in cardiac direct reprogramming, into cardiomyocytes. In this review, we summarize the results of our research on direct cardiac reprogramming over the past decade. Direct cardiac reprogramming is a new method of myocardial regeneration that transdifferentiates cardiac fibroblasts into cardiomyocytes directly within the heart, and we are pursuing research aimed at clinical applications for this new method (Fig. 2).

## Direct reprogramming of fibroblasts into cardiomyocytes

In 2010, we made the novel discovery that cardiomyocytes can be differentiated directly from cardiac fibroblasts, without first being reprogrammed into iPSCs. This differentiation was achieved by introducing three cardiogenic transcription factors: Gata4, Mef2c, and Tbx5 (GMT) [7]. A 2006 report on iPS cells by Yamanaka et al. suggested that multiple organ-specific transcription factors must be introduced to directly reprogram fibroblasts into cardiomyocytes [8]. Ieda et al. created an α-myosin heavy chain (α-MHC)-GFP mouse that expresses the fluorescent protein GFP only in differentiated cardiomyocytes. They established a method for quantitative analysis and screening of cardiomyocyte induction (GFP-positive) from fibroblasts (GFP-negative). They selected fourteen transduction factors that are expressed specifically in cardiomyocytes in the mouse fetus and are important for heart formation and screened them as candidate reprogramming factors. After introducing all 14 genes simultaneously, approximately 1.7% GFP-positive cells were observed after 1 week, indicating the presence of direct cardiac reprogramming factors. The number of candidate factors was reduced from the initial 14 to three cardiogenic transcription factors, Gata4, Mef2c, and Tbx5: GMT, which were essential for direct cardiac reprogramming. Introducing these three factors into cardiac fibroblasts resulted in approximately 17% of GFP-positive cells, subsequently named induced cardiomyocytes (iCMs). Around 4 weeks after induction, the iCMs developed a striation pattern characteristic of cardiomyocytes, and some iCMs developed a spontaneous beat. The iCMs were shown to possess physiological characteristics that closely resemble cardiomyocytes. This characterization was based on the presence of α-actinin, cardiac troponin T (cTnT), and other cardiogenic proteins in these iCMs and the presence of an action potential and periodic changes in Ca^2+^ concentration that were identical to those of mouse neonatal cardiomyocytes.

Recent research has revealed details of the characteristic features of iCMs. Gene expression in iPSC-derived cardiomyocytes (iPSC-CMs) and iCMs of the same mouse were compared, and it was found that the maturation status of iCMs closely resembled that of cardiomyocytes [9]. iPSC-CMs exhibited an active cell cycle and a metabolic profile that indicated glycolysis was the primary means of energy production. The iCMs exhibited a metabolic profile that indicated fatty acid oxidation as the primary means of energy production, similar to adult cardiomyocytes. iCMs also exhibited repression of cell cycle-related genes. These findings suggest that iCMs generated by direct reprogramming possess the potential to advance the field of cardiovascular medicine, not just in terms of regenerative medicine, but also in terms of disease models and drug screening. Cardiac diseases include diverse disorders such as ischemic heart disease, hereditary cardiomyopathy, and metabolic heart disease. Individualized/personalized medicine is currently the focus of attention in cardiology. In the future, myocardial direct reprogramming technology may be used to discover effective drugs for each type of heart disease. In the future, direct cardiac reprogramming technology may be used to discover effective drugs for each type of heart disease. In the case of ischemic heart disease, it is expected that the technology can be used as it is in theory. In hereditary heart disease, gene editing technology and direct cardiac reprogramming can be used for heart regeneration.

## Direct cardiac reprogramming in vivo

The discovery of the direct cardiac reprogramming factor GMT enabled the induction of cardiomyocytes inside the body. Multiple research groups, including our research group, have since reported direct cardiac reprogramming in vivo [10–12]. Each of these studies created a myocardial infarction mouse model using coronary artery ligation and introduced genes into the fibroblasts by injecting a retroviral vector directly into the infarction site. The authors created individual viral vectors for each of the three GMT factors, creating an equal mixture of these three vectors, introduced them into the mouse model, and verified iCM induction in vivo. These iCMs were immature, and the efficiency of induction was poor. The mixing of three separate vectors was assumed to be the reason for this inadequate reprogramming, and therefore, the authors developed a polycistronic vector (3F2A) that combined the three factors on a single vector, allowing them to be introduced uniformly into the fibroblasts. This system improved the efficiency of cardiomyocyte induction twofold compared to the use of individual viral vectors, and a striation pattern was observed in approximately 30% of iCMs at the infarction site [10]. The direct induction of cardiomyocytes from cardiac fibroblasts by introducing GMT genes into the fibroblasts of transgenic mice allowed for fibroblast tracking. This direct in vivo cardiac reprogramming improved cardiac function (left ventricular systolic performance) by approximately 10% after myocardial infarction and resulted in a significant reduction in fibrotic tissue [11]. Song et al. reported that including Hand2 with GMT and introducing genes for these four factors (GMHT) improved the efficiency of in vivo reprogramming and a corresponding improvement in cardiac function was observed [12], similar to that reported by Qian et al. [11]. Based on these reports, direct cardiac reprogramming seems achievable in vivo and it has the potential to become a novel strategy for cardiac regeneration therapy. As detailed later in this chapter, research is underway to develop new vectors that are safe for the host, and we look forward to evaluating their safety in large animals.

## Direct reprogramming of human fibroblasts into cardiomyocytes

After successfully inducing cardiomyocytes by direct reprogramming in vivo, research was initiated into the direct reprogramming of human fibroblasts into cardiomyocytes for clinical application. Our group investigated the efficiency of inducing cardiomyocytes in humans using the same method used in mice. We found that GMT factors sufficient for cardiomyocyte induction in mice were inadequate for cardiomyocyte induction in humans. We screened for new cardiomyocyte induction factors and discovered that two cardiomyocyte/cardiac precursor cell-specific transcription factors, Mesp1 and Myocd, were required in addition to GMT for inducing cardiomyocytes from human cardiac fibroblasts [13]. Human iCMs generated by these five factors—GMT plus Mesp1 and Myocd—developed a striation pattern and expressed cardiomyocyte-specific proteins, but the human iCMs did not exhibit a spontaneous beat. However, when co-cultured with rat cardiomyocytes, these human iCMs started beating in concert and exhibited an action potential specific to cardiomyocytes. In other studies, direct cardiac reprogramming of human cardiac fibroblasts required a combination of six factors (Gata4, Tbx5, Hand2, Myocd, miR-1, and miR-133) [14] or seven factors (Gata4, Mef2c, Tbx5, Mesp1, Myocd, Esrrg, and Zfpm2) [15]. However, each of these research groups reported a lower cardiomyocyte induction efficiency compared to that achieved in mouse fibroblasts and had difficulty inducing functional beating cardiomyocytes.

The genetic basis of the difficulties encountered with direct cardiac reprogramming of human fibroblasts is not well understood. Therefore, single-cell RNA sequencing (scRNA-seq) was used to analyze the reprogramming process in detail [16]. scRNA-seq was previously used to analyze iCM induction from mouse fibroblasts. The key genes for induction were identified, and iCM induction was found to be inhibited in proliferating fibroblasts with an active cell cycle [17]. When direct cardiac reprogramming is compared in mice and humans, iCM induction is repressed in proliferating fibroblasts in both humans and mice, but direct cardiac reprogramming progresses more slowly in humans than in mice. This finding is in line with previous studies that failed to induce beating cardiomyocytes. Inflammation and immune responses hinder the reprogramming process in mice, and epigenetic modifiers including TET1 (ten-eleven translocation methylcytosine dioxygenase 1) are involved in direct cardiac reprogramming in humans [16]. Therefore, in addition to an exhaustive investigation of induction conditions, a more detailed elucidation of molecular mechanisms using methods such as scRNA-seq is vital for progress towards clinical applications. We intend to study the molecular mechanisms of direct cardiac reprogramming and make improvements to direct reprogramming.

## Determining the molecular mechanisms of direct cardiac reprogramming and improving induction efficiency

Direct cardiac reprogramming was a pioneering discovery when it was first reported in 2010, but the cardiomyocyte induction efficiency at the time was low and needed improvement. However, since studies on direct cardiac programming were first published, various approaches have been explored to improve its efficiency. These approaches can be broadly divided into (1) improving direct cardiac reprogramming factors, (2) improving cell culture conditions, and (3) regulating epigenetic factors. Previously unknown factors that inhibit reprogramming have been identified, and next-generation sequencing has elucidated the role of these individual factors in the reprogramming process.

### Improving direct cardiac reprogramming factors

microRNA (miRNA) has been explored as an alternative approach to improving induction efficiency by including transcription factors with GMT, such as GHMT and GMT + Mesp1 and Myocd. miRNAs are small RNA molecules of approximately 21–25 bases that suppress translation by binding to target messenger RNA (mRNA) and play an important role in determining cell fate. Jayawardena reported that directly inoculating mouse hearts with a lentiviral vector hosting four cardiogenic miRNAs (miR-1, miR-133, miR-208, and miR-499) after myocardial infarction directly reprogrammed cardiac fibroblasts into cardiomyocytes and improved cardiac function [18]. We reported that adding these four miRNAs individually and simultaneously to GMT resulted in faster and more efficient cardiomyocyte induction, especially with miR-133 added to GMT, compared to treatment with GMT alone. In human fibroblasts, adding miR-133 to the human cardiomyocyte induction factor GMTMM improved the reprogramming efficiency by approximately 10-fold. miR-133 represses fibroblastic genes from an early point in iCM induction. miR-133 represses Snai1, a key gene in this pathway and a master regulator of the epithelial-to-mesenchymal transition [19]. This discovery established that iCM induction can be enhanced by suppressing fibroblastic traits and laid the groundwork for numerous later discoveries.

### Investigating culture conditions that enhance direct cardiac reprogramming

Direct reprogramming of fibroblasts into iCMs requires the elimination of fibroblast traits. Zhao et al. found that intracellular and extracellular signaling pathways work to maintain the functions of a cell, and in the case of fibroblasts that possess high chemotactic ability and proliferation potential, TGF-β (transforming growth factor-β), Wnt, and ROCK (Rho-associated coiled-coil-containing protein kinase) cytokines are involved in activating their chemotactic ability and proliferation potential. Direct cardiac programming is enhanced by inhibiting the TGF-β and ROCK signaling pathways with low-molecular-weight molecules. Inhibiting the TGF-β and ROCK pathways significantly improves reprogramming efficiency and also allows for the earlier formation of iCMs with a spontaneous beat [20]. Among the 5500 compounds screened for identifying signaling pathways that enhance the efficiency of direct cardiac reprogramming, inhibitors of TGF-β and Wnt signals improved the efficiency of induction with GMT [21].

Signaling pathways that are related to cardiac development and cardiac hypertrophy also enhance direct cardiac reprogramming. Eliciting Akt1 overexpression by adding Akt1 to GHMT resulted in enhanced cardiac reprogramming efficiency and the induction of mature cardiomyocytes [22]. Cardiac reprogramming was also enhanced by repressing Notch signaling. Mef2c transcription is suppressed by Notch signaling, and the addition of DAPT ((S)-tert-butyl 2-((S)-2-(2-(3,5-difluorophenyl) acetamido) propanamido)-2-phenylacetate), a Notch signaling pathway inhibitor, enhanced direct cardiac reprogramming [23]. Our group also attempted to optimize culture media for direct cardiac reprogramming by adding cell growth factors and other cytokines to serum-free media. A combination of fibroblast growth factor (FGF)-2, FGF-10 and vascular endothelial growth factor (VEGF: FFV) enhanced the induction efficiency of beating iCMs approximately 40-fold compared to conventional culture methods. The mechanism behind this effect was the activation of the PI3K/Akt and p38MAPK pathways by cell growth factors, which upregulated the gene clusters involved in cardiac function [24].

### Enhancing direct cardiac reprogramming by regulating epigenetic factors

Cells regulate gene transcription and decide the fate of cell differentiation by DNA methylation and histone modification during the differentiation process. The epigenetics of target genes are also presumed to change substantially during the process of direct reprogramming of fibroblasts into cardiomyocytes. In 2010, we reported that introducing GMT into fibroblasts caused the demethylation of Nppa and Myh6, cardiogenic genes that are methylated in fibroblasts. Histone H3 lysine 4 trimethylation (H3K4me3: active modification) and histone H3 lysine 27 trimethylation (H3K27me3: repressive modification) are two of the most widely recognized histone modifications in cardiogenic and fibroblastic genes [25]. Introducing GMT into fibroblasts increased H3K4me3 and reduced H3K27me3 in cardiogenic gene regions, but decreased H3K4me3 and increased H3K27me3 in fibroblastic gene regions. GMT together with short hairpin RNA (shRNA) for genes involved in histone modifications was introduced into fibroblasts and assessed for epigenetic factors that regulate direct cardiac reprogramming. Bmi1, a component of a polycomb protein complex (PRC1) that causes mono-ubiquitination of histone H2A on lysine 119 (H2AK119ub: repressive modification), was found to epigenetically inhibit the expression of cardiogenic genes. Reducing Bmi1 expression altered the histone modification of cardiogenic genes, resulting in a less repressed state. This modification enhanced the reprogramming efficiency, suggesting that Bmi1 is an epigenetic barrier to reprogramming [26].

Pioneer transcription factors have attracted much attention because they provide important insights into the mechanisms responsible for binding to nucleosomes and subsequent transcriptional regulation [27]. In addition, finding new pioneer transcription factors in cardiac direct reprogramming may help to gain efficiency.

### Age- and inflammation-related suppression of direct cardiac reprogramming

Fibroblastic features and epigenetics have both been identified as barriers to direct cardiac reprogramming. Older neonatal or adult fibroblasts also transform into cardiomyocytes less efficiently than immature embryonic fibroblasts [20, 28]. The underlying mechanism is unknown, and clinical applications would favor efficient cardiomyocyte induction from neonatal or adult fibroblasts. We therefore searched for compounds that enhance cardiomyocyte induction from these fibroblasts.

A library of 8400 chemical compounds was screened for compounds that enhance cardiomyocyte induction, and four compounds that enhance cardiomyocyte induction from neonatal fibroblasts were identified. Further screening revealed that cardiomyocyte induction was enhanced significantly by diclofenac, commonly known as a non-steroidal anti-inflammatory drug (NSAID), to GHMT [29]. Adding diclofenac to GHMT upregulated the expression of cardiogenic proteins and created a distinct striated muscle pattern specific to the heart. Adding diclofenac also increased the number of cardiomyocytes that exhibit spontaneous beating, a characteristic feature of more mature cardiomyocytes, by approximately fourfold. This observation indicated that diclofenac not only enhances cardiomyocyte induction, but also promotes the maturation of the induced cardiomyocytes.

**Fig. 3:**
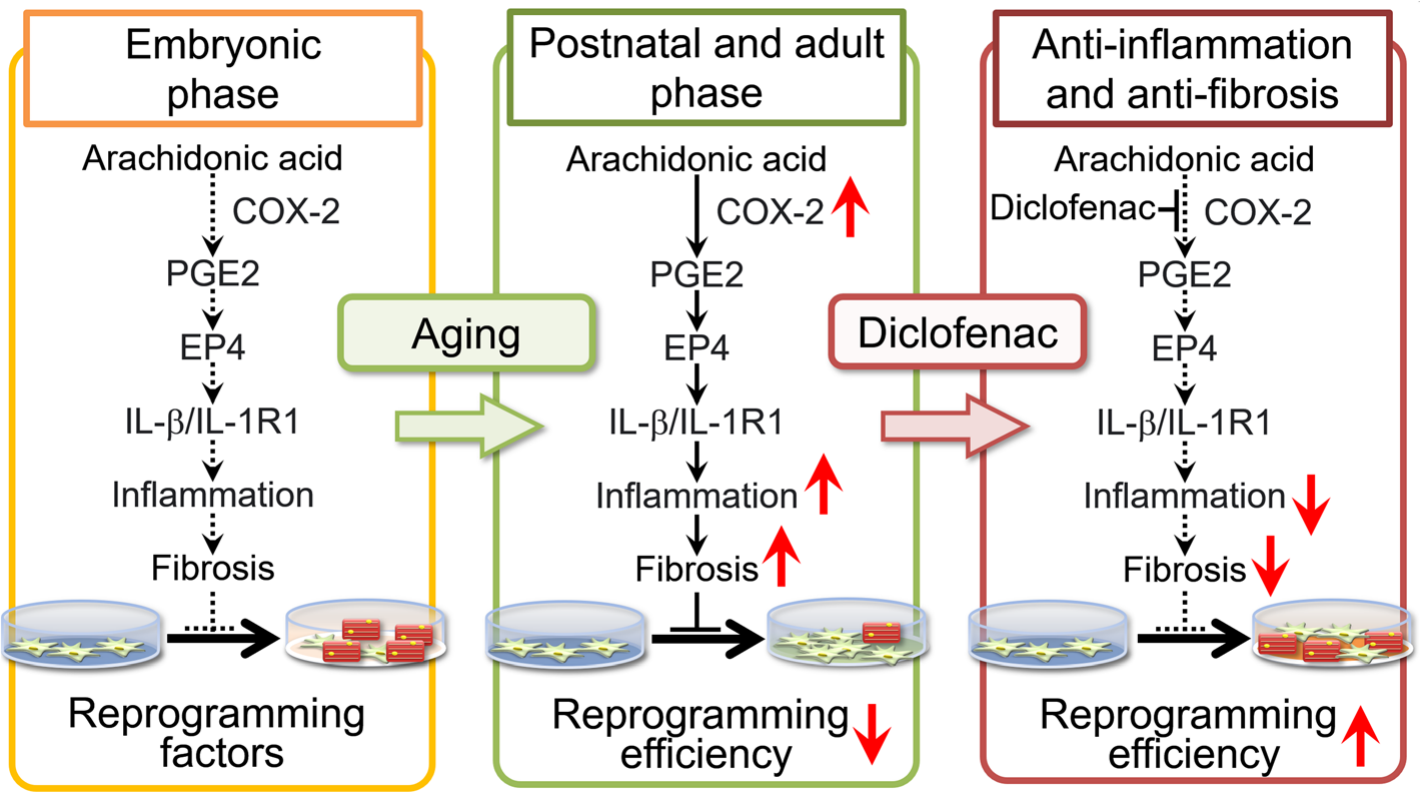
Age-related inflammation and fibrosis are barriers to direct cardiac reprogramming [29]. With aging, fibroblasts activate the COX-2/PGE2/EP4/IL-1β/IL-1R1 pathway. These signaling pathways suppress myocardial induction via expression of fibrosis-related genes (left and middle panels). Diclofenac promotes myocardial induction by suppressing this pathway (right panel)

The enhancement of cardiomyocyte induction by diclofenac was specific to neonatal and adult fibroblasts and was absent in more immature fetal fibroblasts. Fibroblast gene expression changes with age, and there is an increased signaling of cyclooxygenase (COX)-2, prostaglandin E2 (PGE2), and PGE2 receptor EP4 in the arachidonate cascade with increasing fibroblast age. This signaling pathway also induced inflammatory and fibrosing genes further downstream via COX-2/PGE2/EP4/IL-1β/IL-1R1 signaling, which suppressed reprogramming-based cardiomyocyte induction. Diclofenac enhances cardiomyocyte induction by repressing this pathway, and age and inflammation act as barriers to reprogramming (Fig. 3).

Wang L et al. found that Beclin1 (Becn1), an autophagy factor, suppressed the induction of iCM, in a pathway unrelated to autophagy. Conversely, deletion of Becn1 resulted in the high efficient induction of iCM from mouse and human fibroblasts [30].

### The role of transcription factors in direct reprogramming

The quantitative balance among the transcription factors introduced during direct cardiac reprogramming has a major effect on the efficiency of cardiomyocyte induction. Wang et al. reported that high levels of Mef2c expression and low levels of Gata4 and Tbx5 expression were important for enhanced cardiac reprogramming [31]. Next-generation sequencing has enabled the elucidation of the role of each transcription factor in the direct reprogramming process. Hashimoto et al. revealed that fibroblastic genes were repressed, and cardiogenic genes were induced, from early stages of the reprogramming process, using ChIP-seq analysis of the enhancer regions of fibroblastic and cardiogenic genes during reprogramming with GMT [32]. These enhancer regions contain a high frequency of Mef2c binding sites, and the Gata4 and Tbx5 remaining at the Mef2c binding sites probably induce and regulate cardiogenic genes synergistically from early in the reprogramming process. Adding Hand2 and Akt1 results in binding at new enhancer regions, in addition to the previous enhancer regions, and therefore enhances the efficiency of induction [32]. Stone and colleagues reported that Mef2c and Tbx5 caused epigenetic changes between 24 and 48 h after the start of direct cardiac reprogramming by GMT, early in the reprogramming process [33]. Future advances in analytical technology will help to elucidate the molecular mechanisms related to reprogramming, and enable further improvements in direct programming.

### The role of mechanobiology in direct reprogramming

Mechanobiology is believed to be involved in cardiac embryology and cardiac diseases. However, its role in direct cardiac reprogramming remains to be elucidated. The extracellular environment is a key factor in the success or failure of reprogramming. The polystyrene culture dishes in which cells are cultured ex vivo have a hardness of 1 GPa (= 1 × 10^6^ kPa), which is harder than the cartilage tissue (~ 100 kPa) in vivo. The hardness of the heart in vivo is around 10 kPa, but fibrotic scar tissue caused by myocardial infarction is 20–50 kPa. It has been reported that the microenvironment may affect the differentiation of mesenchymal stem cells [34].

Cardiomyocytes derived from iPS cells show enhanced cardiomyocyte maturation upon external stretch stimulation [35]. It has been reported that the reprogramming efficiency of iCMs can be enhanced by seeding fibroblasts on a substrate with microgrooves and transducing reprogramming factors [36]. Mechanotransduction via the extracellular matrix may also induce changes in the chromatin status and affect gene expression. It has been reported that the efficiency of miRNA-mediated direct myocardial reprogramming increases when cells are cultured in a three-dimensional hydrogel, compared to culturing in a two-dimensional culture [37].

**Fig. 4:**
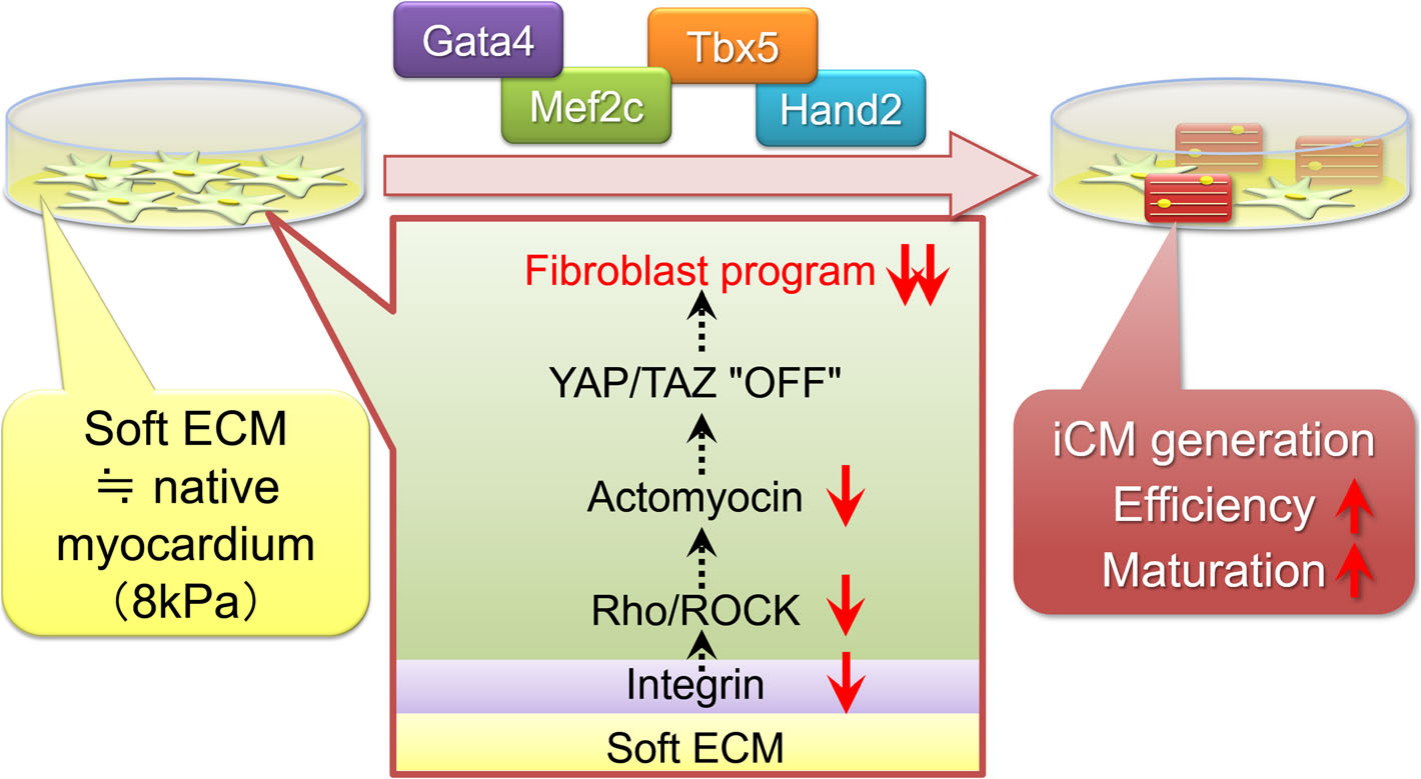
Soft matrix promotes the efficiency and maturation of direct cardiac reprogramming [38]. Soft matrix enhanced cardiac reprogramming via YAP/TAZ suppression. As a result, this can increase the efficiency of induction and maturation of iCMs

We developed a new hydrogel culture system which reproduces the stiffness of myocardial tissue (10 kPa) in vitro. The number of beating iCMs (functional iCMs) increased threefold in the soft hydrogel culture dishes compared to regular polystyrene dishes. We found that YAP/TAZ suppression is involved in soft ECM-mediated cardiac reprogramming. In contrast, the hard dishes increased YAP/TAZ signaling and fibroblast programming, which inhibits direct reprogramming into CMs [38] (Fig. 4). This study is the first report to describe mechanotransduction of matrix stiffness during direct cardiac reprogramming [38].

## Front-line of direct cardiac reprogramming for clinical applications

Research into direct cardiac reprogramming is constantly progressing, although there are limitations to its clinical application, the primary objective. One of these limitations is the development of viral vectors that are safe to use in humans. Retroviral vectors or lentiviral vectors are commonly used to introduce genetic material, but these viruses can potentially damage the host cell genome when introducing multiple genes.

We have created Sendai virus vectors for use in direct cardiac reprogramming [39]. Sendai virus vectors are safe for the simultaneous expression of GMT and other factors. This vector has the advantage of being a non-integration vector and therefore does not damage the host cell genome. Sendai viral vectors improved the efficiency of induction of beating in iCMs by around 100-fold in vitro, compared to previous methods based on retroviral vectors. One of the reasons for this improvement is greater efficiency of protein expression from genes introduced using the Sendai viral vector, compared to those introduced using retroviral vectors. We infected Sendai virus vectors directly into mouse hearts following myocardial infarction and found that cardiac reprograming started after 1 week, and cardiac function improved after 1 month of gene delivery. In vivo, the Sendai virus vectors mainly infected cardiac fibroblasts, the target cells for cardiac reprogramming, and did not infect cardiomyocytes [39].

Like cardiomyocytes, iCMs are terminally differentiated cells with no replication potential. At current induction efficiencies, they are incapable of providing the number of cardiomyocytes needed for clinical applications. During embryonic development, the heart is derived from cardiac mesodermal cells that have the potential for self-renewal and the capacity to differentiate into all cardiac cell types. If fibroblasts could be transformed into cardiac mesoderm, they may differentiate into cardiomyocytes, vascular endothelial cells, and smooth muscle cells. Tbx6 is a transcription factor which is widely expressed in the nascent mesoderm. We revealed that the overexpression of Tbx6 in fibroblasts and PSCs induces direct differentiation into cardiac mesodermal-like cells that express the cardiac mesodermal marker Mesp1. The induced cardiac mesodermal cells from human PSCs differentiated into three cardiovascular lineages, including CMs, which beat spontaneously in culture [40].

## Conclusions

Direct cardiac reprogramming induces cardiomyocytes in situ from cardiac fibroblasts, which proliferate following cardiac diseases. This could be a potentially new therapeutic method for regenerating heart tissue without the need for cell transplantation. However, there are three major problems with direct cardiac reprogramming as follows: (1) the reprogramming efficiency must be improved in humans, (2) the efficiency and safety of in vivo reprogramming should be demonstrated in pigs and other large animals before clinical applications, and (3) the molecular mechanisms of direct reprogramming should be investigated more in detail. Direct cardiac reprogramming has so far only been demonstrated in animal models of myocardial infarction. The efficacy of direct cardiac reprogramming must be investigated in models of dilated cardiomyopathy and other chronic heart diseases which require regenerative therapies. We look forward to further advances in cardiac reprogramming research that bring us closer to cardiac regenerative therapy in the next decades.

## Data Availability

Not applicable

## Abbreviations

α-MHC: Alpha-myosin heavy chain
COX: Cyclooxygenase
cTnT: Cardiac troponin T
DAPT: (S)-tert-butyl 2-((S)-2-(2-(3,5-difluorophenyl) acetamido) propanamido)-2-phenylacetate
FGF: Fibroblast growth factor
GFP: Green fluorescent protein
GMHT: Gata4, Mef2c, Tbx5, Hand2
GMT: Gata4, Mef2c, and Tbx5
iCMs: Induced cardiomyocytes
IL-1β: Interleukin 1β
IL-1R1: Interleukin 1 receptor type 1
iPS cells: Induced pluripotent stem cells
iPSC-CMs: iPSC-derived cardiomyocytes
miRNA: MicroRNA
mRNA: Messenger RNA
NSAID: Non-steroidal anti-inflammatory drug
PGE2: Prostaglandin E2
PRC1: Polycomb protein complex
ROCK: Rho-associated coiled-coil-containing protein kinase
scRNA-seq: Single-cell RNA sequencing
shRNA: Short hairpin RNA
TGF-β: Transforming growth factor-β
VEGF: Vascular endothelial growth factor
